# Epidemiology of Rotavirus Gastroenteritis and Rotavirus-Associated Benign Convulsions with Mild Gastroenteritis after the Introduction of Rotavirus Vaccines in South Korea: Nationwide Data from the Health Insurance Review and Assessment Service

**DOI:** 10.3390/ijerph17228374

**Published:** 2020-11-12

**Authors:** Dong Hyun Kim, Yeong Seok Lee, Dong Jun Ha, Min Jun Chun, Young Se Kwon

**Affiliations:** Department of Pediatrics, School of Medicine, Inha University, Incheon 22332, Korea; id@inha.ac.kr (D.H.K.); yslee1016@inhauh.com (Y.S.L.); djha0917@inhauh.com (D.J.H.); mjchun0716@inhauh.com (M.J.C.)

**Keywords:** rotavirus vaccines, rotavirus infections, seizure

## Abstract

Using nationwide data from the Health Insurance Review and Assessment service, we assessed the impact of rotavirus vaccines, introduced in South Korea, in 2007, on changes in the prevalence of factors (age, sex, and geographic location) associated with rotavirus gastroenteritis (RVGE) and rotavirus-associated benign convulsions with mild gastroenteritis (RaCwG). We analyzed health records of children younger than 3 years who visited clinical facilities and were diagnosed with RVGE or RaCwG between 2007 and 2019. The annual mid-year population (MYP) was obtained from the Korean Statistical Information Service. The annual prevalence of RVGE, RaCwG and associated factors were statistically analyzed. Overall, 219,686, and 4032, children were confirmed to have RVGE and RaCwG, respectively. Although the annual prevalence of RVGE decreased significantly, that of RaCwG did not. The annual ratio of RaCwG to RVGE was significantly high. Compared to the prevalence of RVGE, the prevalence of RaCwG was significantly lower in rural areas. The age of RaCwG patients was significantly lower than that of the MYP and that of RVGE patients. The decrease in the number of RaCwG patients after rotavirus vaccination was not as pronounced as the decrease in the number of RVGE patients.

## 1. Introduction

Rotavirus gastroenteritis (RVGE) is a leading cause of acute gastroenteritis worldwide; it has a high hospitalization rate and is responsible for ~120,000 to ~215,000 deaths of children younger than 5 years each year [1,2,3]. Rotavirus is transmitted via the fecal-oral route and typically causes fever, vomiting, diarrhea, and dehydration [4]. Although rotavirus is known to mainly cause enteric symptoms, several investigators have reported central nervous system complications associated with rotavirus infections such as meningitis [5], encephalopathy [6,7], and encephalitis [8].

Benign convulsions with mild gastroenteritis (CwG) were first reported by Morooka [9] as mild gastroenteritis causing afebrile convulsions without severe dehydration, electrolyte imbalance, and hypoglycemia. Since then, Komori et al. [10] reported the following characteristics of CwG: (1) afebrile seizure occurring within 5 days of acute viral gastroenteritis in previously healthy infants and children; (2) absence of moderate or severe dehydration; (3) presence or absence of repeated convulsive seizures for several days; (4) absence of abnormal cerebrospinal fluid analyses results, serum electrolytes, and blood glucose; (5) a good prognosis; and (6) usually caused by RVGE. Rotavirus-associated benign convulsions with mild gastroenteritis (RaCwG) is characterized by a short illness duration; all episodes of clustered seizures typically subside within 24 h of seizure onset [11,12,13,14]. Although CwG has been frequently observed in East Asian countries, including Japan, South Korea, and Taiwan, it has also been observed in the United States and Europe [15,16,17,18]. However, the underlying pathological mechanism of RaCwG is still unclear.

In 2009 the World Health Organization recommended that the rotavirus vaccine should be included in the National Immunization Program (NIP) in all countries [19]. In South Korea, RotaTeq^®^, a pentavalent vaccine, and Rotarix^®^, a monovalent vaccine, were introduced in June 2007 and March 2008, respectively; however, these vaccines have not yet been included in the South Korean NIP. According to a survey conducted by the Korea Centers for Disease Control and Prevention (KCDC), after their introduction, the rotavirus vaccination rates in South Korea were 5.1%, 26.1%, 34.2%, 45.2%, and 85.6% for infants born in 2007, 2008, 2009, 2011, and 2017, respectively [20].

Since the introduction of rotavirus vaccines, the prevalence of RVGE has decreased in South Korea [21,22,23]. However, epidemiological studies of RaCwG after vaccination are lacking. Additionally, no nationwide data regarding the epidemiology of RaCwG are available.

Therefore, in this study, we used data from the Health Insurance Review and Assessment service (HIRA) to assess the impact of rotavirus vaccines based on changes in the prevalence of and factors associated with RVGE and RaCwG in South Korea.

## 2. Materials and Methods

### 2.1. Data Sources

The National Health Insurance is the only compulsory public medical insurance system in South Korea [24], and it includes data regarding more than 98% of the national population [25,26]. The HIRA database provides all diagnostic codes and medical records claimed to researchers. Using the HIRA database, we collected the number of RVGE patients and the number of RaCwG patients who visited clinics or hospitals. In addition, to determine disease prevalence, we obtained the annual mid-year population (MYP) from the Korean Statistical Information Service (KOSIS). This study was approved by the Institutional Review Board of Inha University Hospital (IRB no. 2020-01-036).

### 2.2. Study Population

We assessed health record data of patients younger than 3 years with RVGE and the diagnostic code for ‘rotavirus gastroenteritis’ (International Classification of Disease 10th Revision (ICD-10) code: A08.0) between 2007 and 2019 that were available in the HIRA database. Data for 2019 were only available until May; therefore, data for the years until 2018 were used. The RVGE episode was considered to have ended when there was no RVGE claim for more than 14 days since the last claim. Patients whose records claimed ‘acute gastroenteropathy’ (A08.1), ‘adenoviral enteritis’ (A08.2), ‘sapoviral gastroenteritis’ (A08.30), and ‘astroviral gastroenteritis’ (A08.31) were excluded.

RaCwG patients were included if at an age less than 3 years, their records included A08.0 and ‘convulsions of new born’ (P90), ‘convulsion’ (R56) or ‘other and unspecified convulsions’ (R56.8) in neonates younger than 29 days, and R56 or R56.8 for patients between 29 days and 3 years of age. Based on the criteria of Komori et al. [10], we excluded patients who claimed the following diagnostic codes: (1) ‘febrile convulsions’ (R56.0), (2) ‘dehydration’ (P74, E86), (3) ‘epilepsy’ (G40), (4) ‘meningitis’ (A87, G00, G01, G02, G03), ‘encephalitis’ (B00.4, A83, A84, A85, G04, G05), ‘disorders of fluid, electrolyte and acid-base balance’ (E87), or ‘hypoglycemia’ (E16, P70).

### 2.3. Demographics and Annual Prevalence of RVGE and RaCwG

The annual prevalence of RVGE and RaCwG were calculated using the MYP. The annual ratio of the number of cases of RaCwG to the number of cases of RVGE was obtained. To compare factors that could affect the prevalence of RVGE and RaCwG, we collected the number of patients with RVGE and the number of patients with RaCwG according to sex, age, and different geographical locations (urban areas: Seoul, Busan, Daegu, Incheon, Gwangju, Daejeon, Ulsan, and Sejong; rural areas: Gyeonggi, Gangwon, Chungbuk, Chungnam, Jeonbuk, Gyeongbuk, Gyeongnam, and Jeju).

### 2.4. Statistical Analysis

The data analysis was performed by remote access to the HIRA database and using SAS Enterprise version 9.2 (SAS Institute, Cary, NC, USA). Statistical analyses were performed using SPSS version 19.0 (IBM, Armonk, NY, USA). A negative binomial regression analysis was performed to identify the prevalence trends of RVGE and RaCwG and to identify factors (age, sex, geographical location) that may affect the prevalence. *p* < 0.05 was considered statistically significant.

## 3. Results

### 3.1. Characteristics of Patients with RVGE, Patients with RaCwG, and the MYP

Overall, 219,686 children were confirmed to have RVGE between 2007 and 2018. Among them, 121,110 were male and 98,576 were female. The number of patients with RaCwG was 4032. Among them, 2102 were male and 1930 were female. The MYP was 15,960,271.5. Among them, 8,209,580.5 were male and 7,750,691 were female (Table 1). The total prevalence of RVGE and RaCwG were 13.8% and 0.024%, respectively.

### 3.2. Annual Prevalence of RVGE, Annual Prevalence of RaCwG, and Ratio of RaCwG to RVGE

**Figure 1:**
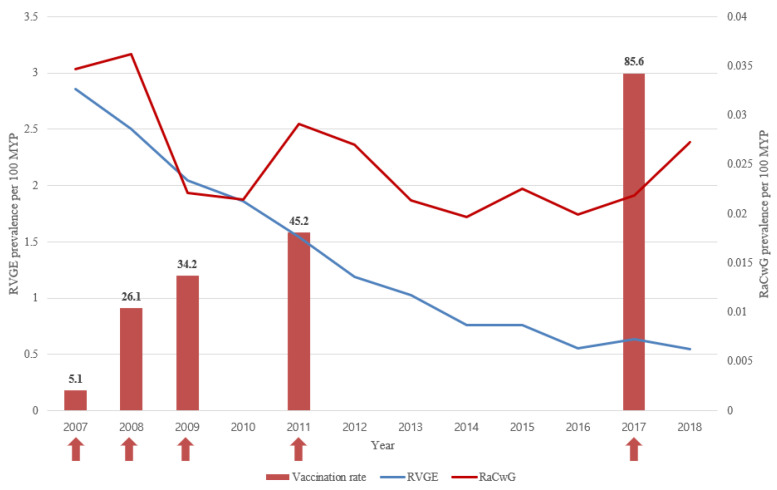
The prevalence of rotavirus gastroenteritis (RVGE) and rotavirus-associated benign convulsions with mild gastroenteritis (RaCwG) and the vaccination rate [18].

As the vaccination rate increased, the prevalence of RVGE significantly decreased by 0.852 times each year (*p* < 0.001). The annual prevalence of RaCwG decreased by 0.941 times each year, but this was not statistically significant (*p* = 0.139) (Figure 1). Furthermore, the annual ratio of RaCwG to RVGE increased significantly by 1.105 times each year (*p* = 0.018).

### 3.3. Associated Factors with RVGE and RaCwG

**Figure 2:**
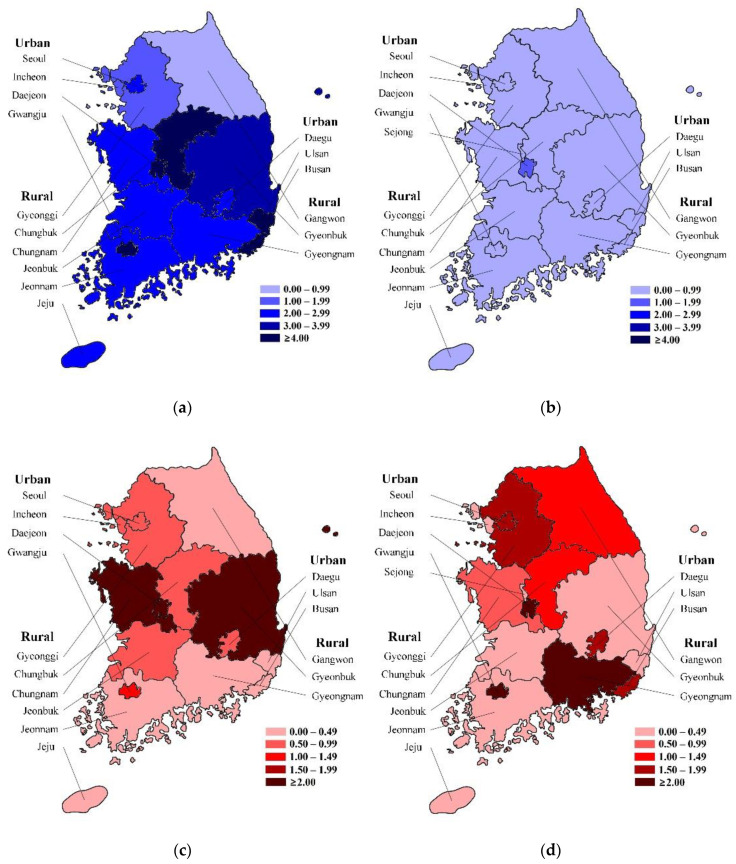
Regional rotavirus gastroenteritis (RVGE) prevalence per 100 individuals comprising the mid-year population (MYP) and the ratio of rotavirus-associated benign convulsions with mild gastroenteritis (RaCwG) to 100 RVGE patients. Darker shades indicate a higher prevalence and a higher ratio. (**a**) RVGE prevalence per 100 comprising the MYP in 2007. (**b**) RVGE prevalence per 100 comprising the MYP in 2018. (**c**) Ratio of RaCwG to 100 RVGE patients in 2007 (**d**) Ratio of RaCwG to 100 RVGE patients in 2018.

In terms of geographic location, the prevalence of RVGE did not demonstrate a significant difference between urban and rural areas (*p* = 0.475). However, the prevalence of RaCwG in rural areas was significantly lower (*p* < 0.001). When compared with the prevalence of RVGE, the prevalence of RaCwG in rural area was lower than that in urban areas (*p* < 0.001). As time progressed, the prevalence of RVGE decreased regardless of the geographic region, but the ratio of RaCwG to RVGE increased, especially in urban areas (Figure 2). In terms of age, the proportion of 2-year-olds children with RVGE was significantly lower than the proportion of 2-year-olds comprising the MYP (*p* = 0.008). The proportion of 2-year-olds with RaCwG was significantly lower than the proportion of 2-year-olds comprising the MYP (<0.001) and the proportion of 2-year-olds with RVGE (*p* = 0.015). There was no statistically significant difference in the sex ratio of patients with RVGE, patients with RaCwG, and the MYP (Table 2).

## 4. Discussion

To determine the epidemiology of RVGE and RaCwG in South Korea, after the introduction of rotavirus vaccines in 2007, this study used nationwide data from the HIRA database and KOSIS. We found that although the prevalence of RVGE decreased since 2007, the prevalence of RaCwG did not. Furthermore, RaCwG was affected by factors such as age and geographic location. RaCwG was diagnosed at a younger age and occurred more often in urban areas than in rural areas compared to RVGE.

Prior to the introduction of the rotavirus vaccine introduction, rotavirus was responsible for 20–60 deaths per year in the United States and up to 500,000 deaths attributable to diarrhea worldwide [27]. In South Korea, before rotavirus vaccination, the annual incidence of rotavirus infection was 56.9 per 1000 children, and the hospitalization rate due to rotavirus infection was 11.6 per 1000 children [28]. Since the introduction of vaccination, the incidence of RVGE has decreased in all regions of South Korea [29,30]. In this study, we also found that the prevalence of RVGE has decreased since 2007. We suspected that these effects are attributable to rotavirus vaccination. Therefore, if rotavirus vaccines are included in the NIP in South Korea, and if the rotavirus vaccination rate increases, then the prevalence of RVGE should decrease. Though the prevalence of RVGE decreased after vaccination, the prevalence of RaCwG did not, which is a strength of this study. Moreover, the ratio of RaCwG to RVGE increased annually. Previous reports showed that the ratio of RaCwG to RVGE has ranged from 1.29% to 5.4% [31,32,33]. A study of the incidence relative to the population was conducted in Taiwan and indicated the incidence of RaCwG was 88.5 per 100,000 children [34]. After vaccination was introduced, there has been only one study involving a single center that indicated that the number of patients with RaCwG decreased in South Korea [35]. In this study, we found that the total prevalence of RaCwG, which was as low as 0.024%, decreased 0.941 times per year. This result could be attributable to rotavirus vaccination, but it was not statistically significant. The prevalence of RaCwG did not decrease statistically in this study, possibly due to several factors that influence its prevalence. 

A recent study found that an elevated level of serum uric acid is a factor for CwG [36]. In this study, we demonstrated that factors such as age and geographic location influenced RVGE and RaCwG differently; therefore, the prevalence of RaCwG is likely affected by various factors. Further studies are necessary to investigate other factors that increase the ratio of RaCwG to RVGE.

In this study, we found no difference in the prevalence of RVGE in urban and rural areas. A previous study conducted in southern Mozambique demonstrated that there was no difference in the prevalence of RVGE among patients reporting enteric symptoms in urban and rural areas [37]; however, no research in South Korea observed differences in the prevalence of RVGE and RaCwG based on geographic location. Globally, the incidence of rotavirus disease is similar for children in developed and developing nations, suggesting that adequate control may not be achieved by improving the water supply, hygiene, and sanitation [38]. In this study, our results also showed that the prevalence of RVGE did not change according to geographic locations, thus supportings previous studies that hypothesized that the prevalence of RVGE is not reduced by improving the water supply, hygiene, and sanitation. However, RaCwG was diagnosed significantly more often in urban areas when compared with the MYP and the diagnostic frequency of RVGE. We suggest that this could have been because there are more tertiary hospitals in urban areas where seizures can be managed. However, further research is needed to determine if there are other environmental factors or socioeconomic factors that cause more cases of RaCwG in urban areas.

After the introduction of vaccines in South Korea, children with RVGE who needed hospitalization were relatively older [21,39]; the mean age of hospitalized patients was between 1 and 2 years [40]. In a previous study, the age of RaCwG patients ranged from 4 months to 3 years [10], with the highest incidence of RaCwG observed in children between 1 and 2 years [41]. In this study, the age of RVGE patients was lower than the MYP. This could be because the severity of RVGE decreases with each repeated infection [42,43,44]; therefore, when older children are infected, there are no symptoms and they do not go to the hospital. The age of patients with RaCwG was significantly lower than that of patients with RVGE. This could be attributable to the vulnerability of the developing central nervous system at younger ages [44].

In this study, there was no difference in the prevalence of males and females. In previous studies, an epidemiological difference between males and females was not observed for RVGE [45,46] and RaCwG [41]. Although the vaccination rate of girls was higher than that of boys in South Korea [20], we suspect that the vaccination rate based on sex was not sufficient to affect the actual rotavirus infection rate. Additionally, sex differences are considered factors that do not affect the occurrence of RaCwG.

This study was limited because it did not compile data by directly reviewing patient charts. Estimating the prevalence of RVGE using the A08.0 ICD-10 code of created the risk of under-estimation [47,48,49]. Furthermore, patients with the diagnostic code A08.0 in their medical records were considered the RVGE group; however, rotavirus was not confirmed by the RNA. Therefore, the actual number of RVGE patients and the number of patients in the RVGE group may have been different.

## 5. Conclusions

RaCwG is affected by factors such as age and geographic location. In addition, Furthermore, the decrease in the number of RaCwG patients after rotavirus vaccination was not as pronounced as the decrease in the number of RVGE patients. Continuous monitoring is needed to determine if the annual ratio of RaCwG to RVGE will increases.

## Figures and Tables

**Table 1:** Characteristics of patients with RVGE, RaCwG, and MYP.

Variable	RVGE	RaCwG	MYP
Subjects, number	219,686	4032	15,960,271.5
Sex			
Male	121,110 (55.1)	2102 (52.1)	8,209,580.5 (51.4)
Female	98,576 (44.9)	1930 (47.9)	7,750,691 (48.6)
Age (years), median (IQR)	1.0 (0.0–1.0)	0.0 (0.0–1.0)	1.0 (0.0–2.0)
Year			
2007	38,301 (17.4)	465 (11.5)	1,340,068 (8.4)
2008	34,226 (15.6)	495 (12.3)	1,366,555 (8.6)
2009	28,213 (12.8)	305 (7.6)	1,382,048 (8.7)
2010	25,467 (11.6)	293 (7.3)	1,369,526.5 (8.6)
2011	21,008 (9.6)	397 (9.8)	1,362,404 (8.5)
2012	16,524 (7.5)	374 (9.3)	1,387,208 (8.7)
2013	14,285 (6.5)	298 (7.4)	1,394,688.5 (8.7)
2014	10,349 (4.7)	268 (6.6)	1,364,246 (8.5)
2015	10,045 (4.6)	298 (7.4)	1,324,410 (8.3)
2016	7147 (3.3)	257 (6.4)	1,288,368 (8.1)
2017	7868 (3.6)	270 (6.7)	1,236,382.5 (7.7)
2018	6253 (2.8)	312 (7.7)	1,144,367 (7.2)

RVGE: rotavirus gastroenteritis; RaCwG: rotavirus-associated benign convulsions with mild gastroenteritis; MYP: mid-year population; IQR: interquartile range. Results are expressed as the number of patients (%) unless otherwise indicated.

**Table 2:** Negative binomial regression analysis of patients with RVGE, patients with RaCwG, and the MYP.

Variable	RVGE/MYP				RaCwG/MYP				RaCwG/RVGE			
	β	Exp(B)	95% CI	*p* Value	β	Exp(B)	95% CI	*p* Value	β	Exp(B)	95% CI	*p* Value
Age (≥2 year)	−1.091	0.336	0.151–0.748	0.008 *	−2.125	0.119	0.053–0.270	<0.001 *	−1.008	0.365	0.162–0.821	0.015 *
Sex (female)	−0.136	0.873	0.496–1.538	0.639	−0.031	0.969	0.548–1.715	0.915	0.100	1.105	0.624–1.956	0.732
Area (rural)	−0.206	0.814	0.462–1.433	0.475	−1.334	0.264	0.147–0.473	<0.001 *	−1.109	0.330	0.185–0.589	<0.001 *
Year	−0.161	0.852	0.786–0.923	<0.001 *	−0.061	0.941	0.867–1.020	0.139	0.099	1.105	1.017–1.199	0.018 *

RVGE/MYP: number of patients with rotavirus gastroenteritis compared with the mid-year population. RaCwG/MYP: number of patients with rotavirus-associated benign convulsions with mild gastroenteritis compared with the mid-year population. RVGE/RaCwG: number of patients with rotavirus-associated benign convulsions with mild gastroenteritis compared with number of patients with rotavirus gastroenteritis.CI: confidence interval. * *p* < 0.05.
